# Structural Polymer-Based Carbon Nanotube Composite Fibers: Understanding the Processing–Structure–Performance Relationship

**DOI:** 10.3390/ma6062543

**Published:** 2013-06-20

**Authors:** Kenan Song, Yiying Zhang, Jiangsha Meng, Emily C. Green, Navid Tajaddod, Heng Li, Marilyn L. Minus

**Affiliations:** Department of Mechanical and Industrial Engineering, Northeastern University, 334 Snell Engineering Center, 360 Huntington Avenue, Boston, MA 02115, USA; E-Mails: song.k@husky.neu.edu (K.S.); zhang.yiyi@husky.neu.edu (Y.Z.); meng.ji@husky.neu.edu (J.M.); green.e@husky.neu.edu (E.C.G.); tajaddod.n@husky.neu.edu (N.T.); li.heng@husky.neu.edu (H.L.)

**Keywords:** carbon nanotubes, polymer, mechanical properties, preparation, synthesis, dispersion, interphase, alignment, applications

## Abstract

Among the many potential applications of carbon nanotubes (CNT), its usage to strengthen polymers has been paid considerable attention due to the exceptional stiffness, excellent strength, and the low density of CNT. This has provided numerous opportunities for the invention of new material systems for applications requiring high strength and high modulus. Precise control over processing factors, including preserving intact CNT structure, uniform dispersion of CNT within the polymer matrix, effective filler–matrix interfacial interactions, and alignment/orientation of polymer chains/CNT, contribute to the composite fibers’ superior properties. For this reason, fabrication methods play an important role in determining the composite fibers’ microstructure and ultimate mechanical behavior. The current state-of-the-art polymer/CNT high-performance composite fibers, especially in regards to processing–structure–performance, are reviewed in this contribution. Future needs for material by design approaches for processing these nano-composite systems are also discussed.

## 1. Introduction

Since the birth of polymer science in the 1930s, these materials have dominated the market in terms of their versatility for product applications. These materials have been utilized in the form of films, fibers, sheets, and coatings. Today, most of the synthetic polymer fibers in use span applications such as clothing, carpets, ropes, and reinforcement materials. Some of these fibers include polyamides such as nylon, polyesters [e.g., polyethylene terephthalate (PET) and polybutylene terephthalate (PBT)], polyolefins [e.g., polypropylene (PP) or polyethylene (PE)], vinyl polymers [e.g., poly(vinyl alcohol) (PVA) and poly(vinyl chloride) (PVC)], elastomers (e.g., polyurethane (PU) and spandex), and acrylic fibers (e.g., polyacrylonitrile (PAN)) [1]. In addition, high-performance polymer-based fibers with high stiffness and/or tenacity include Dyneema^®^ and Spectra^®^ (*i.e.*, ultra-high molecular weight polyethylene (UHMWPE)-based fibers), Twaron^®^ and Kevlar^®^, and Zylon^®^ fibers (*i.e.*, aromatic-based polymers such as poly(*p*-phenyleneterephthalamide) (PPTA) and poly(*p*-phenylenebenzobisoxazole) (PBO)) [2]. Also included is PAN, which is the dominant precursor fiber for the carbon fiber industry. The typical properties for these materials are listed in Table 1.

**Table 1:** Typical mechanical properties’ values for commercially available polymer fibers used for textile and high-performance applications.

Classification of Fibers	Fiber type	Strength (GPa)	Modulus (GPa)	Strain (%)	References
Textile Fibers	Polyamide	0.91	9.57	37	[3]
	Polyesters	0.7 to 0.9	12 to 17	7 to 37	[4]
	Vinyl Fibers	0.3 to 0.66	~4.5 to 7.5	<40	[4,5]
	Elastomers	0.004 to 0.009	0.01 to 0.03	>500	[5]
High-Performance Fibers	Spectra^®^	2.5 to 3.6	97 to 133	2.8 to 4.5	[5]
	Dyneema^®^	3.4	113	3.5	[5]
	Kevlar^®^	1.44 to 3.6	62 to 190	1 to 4.4	[3]
	Zylon^®^	4.2	280	2.5	[3]
	M5	up to 9	330 to 350	1.2 to 1.5	[3,4]
	PAN-based carbon fibers	2.5 to 3.8	227 to 405	0.8 to 1.76	[3,4]

Despite the significant amount of progress made towards producing high-performance fibers from polymer materials, the mechanical properties still remain only a fraction of the expected theoretical values for these materials. Several technological developments in recent years have been used to improve the high-performance properties of polymer-based fibers. For example, manufacturing processes by sea/island composite-spinning technology have progressed to produce commercial nano-fibers with ~700 nm uniform diameters and high tenacity [6,7]. Commercial products, including functional sportswear (*i.e.*, golf gloves), inner wear, skin-care products, filters, and precision grinding cloths (*i.e.*, polishing cloths), taking advantage of these polyester nano-fibers have been developed due to the large surface area, high adsorption, good dispersion, and filtration effects belonging to these thin fibers [6].

Other opportunities to produce high-performance polymer materials include using fillers to produce composites. Carbon fiber and glass fiber composites were first produced in the 1960s and 1970s, leading to disruptive technological evolutions in the field of material science [8,9,10,11]. The first polymer composites (*i.e.*, fiber glass) revolutionized the boating industry, and later in the 1960s, the advent of carbon fibers ushered in many disruptive technologies for producing polymer composites and increasing their applications. Since that time, carbon-fiber-reinforced polymer composites (CFRP) have remained a major standard for polymer-based materials in high-performance applications. Currently, the carbon fiber (CF) market is dominated by the U.S. and Japan, where production is expected to increase to 80,000 tons by 2016 [12]. The use of CF materials has increased at an average annual rate of ~12% for the last 23 years, and this has been attributed to the development of new energy technologies (e.g., wind energy) and industrial applications requiring lightweight materials [13]. The one major hindrance of CF usage has been the high cost, and for that reason ~47% of the CF usage is within the aerospace sector. Pseudo one-dimensional fibers such as aluminum, glass, boron, silicon carbide, and carbon nano-fibers (CNF) have also been used over the years as fillers in composites. Typical composite stiffness and strength properties range from 230 to 725 GPa and 1.5 to 4.8 GPa, respectively [14].

The recognition of multi-wall carbon nanotubes (MWNT) in 1991 [15] and single-wall carbon nanotubes (SWNT) in 1993 [16] brought about a new influx of research in lightweight high-performance reinforced polymers. As compared to the conventional carbon fibers, Young’s modulus and tensile strength of these tubular graphitic materials have subsequently been measured to be ~1 TPa [17,18,19,20] and ~10 to 150 GPa [21,22,23,24], respectively. Therefore, composites incorporating carbon nanotubes (CNT) have received a great deal of attention in both academia and industry for their potential replacement of carbon fibers in polymer-based reinforced materials. Several reviews have already focused on summarizing the property enhancement of polymers by CNT [25,26,27,28,29,30,31,32]. CNT have been heralded as a game changer for producing next-generation high-performance materials that would trump the properties of current CFRPs. However, one major hangup has also been the cost of these materials at small-scale production levels. One potential route toward reducing the cost of the CNT-based composites is through using small quantities of CNT to reinforce the polymer for high-performance applications.

A brief listing of the typical property improvements in polymer/CNT fibers is provided in Table 2. To date, the best polymer/CNT fibers have tensile strengths ranging from ~0.1 to 5 GPa and modulus values from ~5 to 200 GPa. However, in terms of repeatability for fabricating these composites, the typical tensile strength values range from 0.5 to 2 GPa [33]. Structural composite fibers are of great importance for several industrial uses including, but not limited to, automotive, aerospace, consumer products, transportation, and construction [34]. Additionally, due to the unique combination of properties, usage of CNT in polymer composites not only improves strength and modulus but can also result in enhancements in chemical resistance, thermal conductivity, electrical conductivity, and dimensional stability [34]. For this reason, fundamental understanding for producing new high-performance materials is necessary. This contribution will focus mainly on the relationship between polymer/CNT fiber fabrication methods and the micro-structural development during processing. These fundamental issues are significant and need to be addressed for material design toward commercialization of polymer-based CNT composite fibers meant for high-performance technologies.

**Table 2:** Summary of the typically reported mechanical properties for polymer-based carbon nanotube composite fibers.

References	Sample (polymer + wt % CNT)	Mechanical properties			
		Elastic modulus [GPa]	Strength [GPa]	Strain [%]	Toughness
[35,36]	Poly(vinyl alcohol) (PVA) + >60 wt % SWNT	9 to 15	0.15	~3	–
	PVA + ~60 wt % SWNT	80	1.8	>100	570 J·g^−1^
[37]	PVA + ~60 wt % SWNT	40	0.3	>400	600 J·g^−1^
[38]	Commercial PVA fiber	40	1.6	7	–
	PVA + >60 wt % SWNT	78	1.8	~40	120 ± 152 J·g^−1^
[39]	PVA + 2–31 wt % SWNT	Up to 244	Up to 2.9	~3–10	–
[40]	PVA	21.8 ± 3.0	1.2 ± 0.3	11.4 ± 1.7	55.8 ± 12.3 J·g^−1^
	PVA + 10 wt % SWNT	36.3 ± 1.3	2.5 ± 0.1	10.7 ± 0.7	101.4 ± 11.4 J·g^−1^
	PVA + 10 wt % SWNT	119.1 ± 8.6	4.4 ± 0.5	9.7 ± 1.1	171.6 ± 30.4 J·g^−1^
[41]	PVA	~13	~0.4	~15	–
	PVA + 1 wt % SWNT	~17.5	~1.2	~17.5	–
[42]	PVA	45 ± 7	1.0 ± 0.1	5.3 ± 0.3	22 ± 4 J·g^−1^
	PVA + 1 wt % SWNT	60 ± 6	1.4 ± 0.1	4.9 ± 0.5	29 ± 6 J·g^−1^
	PVA	48 ± 3	1.6 ± 0.1	6.5 ± 1.4	40 ± 6 J·g^−1^
	PVA + 1 wt % SWNT	71 ± 6	2.6 ± 0.2	6.2 ± 0.7	59 ± 7 J·g^−1^
[43]	Polyacrylonitrile (PAN)	22.1 ± 1.2	0.90 ± 0.18	7.4 ± 0.8	35 ± 9 MPa
	PAN + 0.5 wt % SWNT	25.5 ± 0.8	1.06 ± 0.14	7.2 ± 0.6	41 ± 8 MPa
	PAN + 1 wt % SWNT	28.7 ± 2.7	1.07 ± 0.14	6.8 ± 0.8	39 ± 8 MPa
[44,45]	Carbonized PAN	302 ± 32	2.0 ± 0.4	0.68 ± 0.04	–
	Carbonized PAN + 1 wt % SWNT	450 ± 49	3.2 ± 0.4	0.72 ± 0.05	–
[46]	Poly(*p*-phenylenebenzobisoxazole) (PBO)	138 ± 20	2.6 ± 0.3	2.0 ± 0.2	−
	PBO+>60 wt % SWNT	167 ± 15	4.2 ± 0.5 (~50% increase)	2.8 ± 0.3	−
[47]	Polypropylene (PP)	6.3	0.71	18.9	7.93 dN/tex
	PP + 0.5 wt % SWNT	9.3	0.84	19.1	9.37 dN/tex
	PP + 1 wt % SWNT	9.8	1.03	26.6	11.5 dN/tex
[48]	Nylon 6	0.44	0.045	−	−
	Nylon 6 + 0.1 wt % SWNT	0.54	0.086	−	−
	Nylon 6 + 0.2 wt % SWNT	0.66	0.093	−	−
	Nylon 6 + 0.5 wt % SWNT	0.84	0.083	−	−
	Nylon 6 + 1.0 wt % SWNT	1.15	0.083	−	−
	Nylon 6 + 1.5 wt % SWNT	1.2	0.075	−	−
[49]	Ultra-high molecular weight polyethylene (UHMWPE)	2.42 ± 0.40	0.11 ± 0.002	402.0 ± 20.1	361.8 ± 22.9 MPa
	UHMWPE + 5 wt % MWNT	2.62 ± 0.32	0.13 ± 0.004	540.4 ± 104.7	593.2 ± 114.5 MPa
	UHMWPE	122.6 ± 1.9	3.51 ± 0.13	4.03 ± 0.15	76.7 ± 7.5 MPa
	UHMWPE + 5 wt % MWNT	136.8 ± 3.8	4.17 ± 0.04	4.65 ± 0.35	110.6 ± 10.5 MPa

## 2. General Fabrication Procedures for Polymer/CNT Fibers

In general, when discussing polymer/CNT composites, two major classes come to mind. First, the CNT nano-fillers are dispersed within a polymer at a specified concentration, and the entire mixture is fabricated into a composite. Secondly, as-grown CNT are processed into fibers or films, and this macroscopic CNT material is then embedded into a polymer matrix [50]. This review paper will focus on the first class of polymer/CNT composite materials to explore their processing–structure–property relationships.

**Figure 1:**
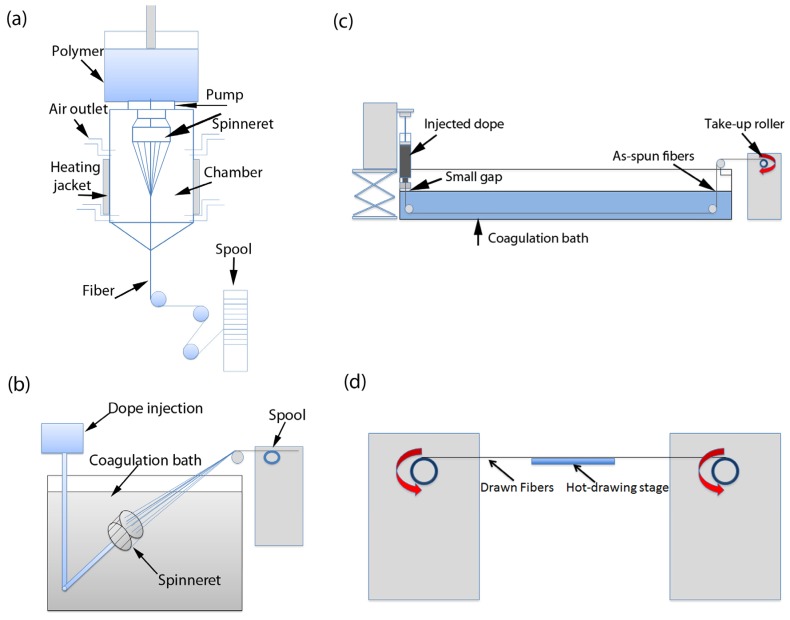
Schematics representing the various fiber processing methods (**a**) dry-spinning; (**b**) wet-spinning; (**c**) dry-jet wet or gel-spinning; and (**d**) post-processing by hot-stage drawing.

The four major fiber-spinning methods (Figure 1) used for polymer/CNT composites from both the solution and melt include dry-spinning [51,52], wet-spinning [53], dry-jet wet spinning (e.g., gel-spinning [54]), and electro-spinning [55,56]. An ancient solid-state spinning approach has been used for fabricating 100% CNT fibers from both forests and aerogels [57,58,59,60]. Regardless of the processing technique, in order to develop high-quality fibers many parameters need to be well controlled. In general, all spinning procedures involve (i) fiber formation; (ii) coagulation/gelation/solidification; and (iii) drawing/alignment. For all of these processes, the even dispersion of the CNT within the polymer solution or melt is very important. However, in terms of achieving excellent axial mechanical properties, alignment and orientation of the polymer chains and the CNT in the composite is necessary. Fiber alignment is accomplished in post-processing such as drawing/annealing and is key to increasing crystallinity, tensile strength, and stiffness [61].

Alignment in polymer/CNT composite fibers is dependent on the polymer chain conformation, CNT morphology, and dispersion in the matrix. In terms of polymer conformation, for linear flexible polymers achieving high orientation and extension requires uncoiling and disentanglement of the chains. Very stiff polymer rod-like chains (e.g., aromatic polymers) are able to self-assemble and form aligned structures during processing. In this way, the fiber microstructure can be very different and this translates to the composite morphology. The CNT morphology and dispersions are also important, since these have influences on the polymer structural development. The processing of these composites has a direct effect on the ultimate structure–property relationship for polymer/CNT fibers.

## 3. Micro-Structural Development in Polymer/CNT Fibers

**Figure 2:**
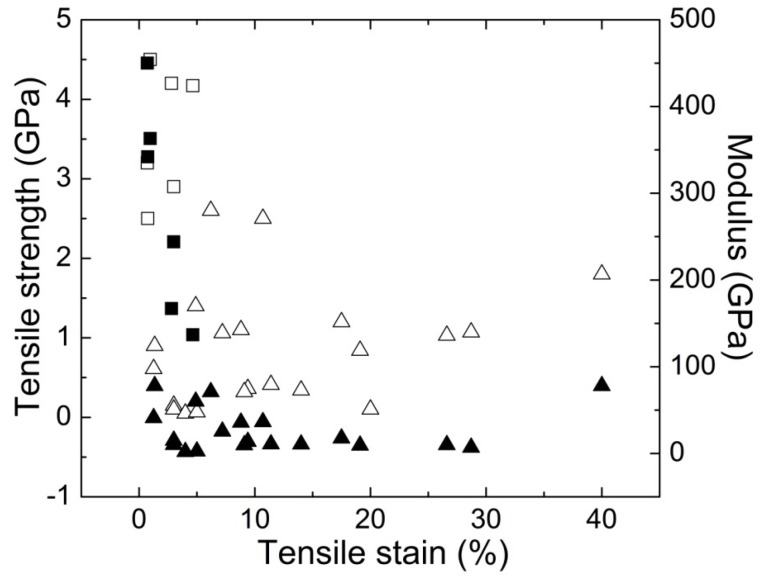
Summary of Young’s modulus, tensile strength, and strain-to-failure properties for various polymer/CNT fibers produced at the research scale which exhibit properties similar to high-performance commercial fibers [35,36,38,39,40,41,42,43,44,45,46,47,112,113,114,115,116] (Note: □/■ symbols represent tensile strength/modulus properties for high-performance fibers, and ∆/▲ symbols represent tensile strength/modulus properties for textile-grade fibers).

The overall picture of mechanical performance for polymer/CNT fibers produced at the research level shows a broad range of properties (Figure 2). These fibers were produced using several fabrication methods. As mentioned, the discovery of CNT ushered in a large amount of research efforts focused on utilizing these nano-materials to make polymer composite fibers to capture these exceptional properties (*i.e.*, 1 TPa in tensile modulus and 10 to 150 GPa [21,22,23,24] in tensile strength). However, this realization has been lacking in spite of there being an additional host of nano-carbon materials produced since their discovery (*i.e.*, SWNT, MWNT, and vapor-grown carbon nano-fibers (VGCNF), as well as layered graphitic materials). This disappointment has led to a decline in the hype surrounding such composites and a shift of research focus to some of the other unique features of these materials such as their electrical [57,62,63,64,65,66,67,68,69,70,71,72,73,74,75,76,77,78,79,80,81,82,83,84,85,86,87,88,89,90,91,92,93], thermal [57,68,73,76,86,94,95,96,97,98,99,100,101,102,103], and optical properties [104,105,106,107,108,109]. This rise and fall of interest in polymer nano-composite research is analogous to what happened between 1832 and 1939 when polymers were originally discovered but not well understood [110,111]. As a result, these materials (polymers) went underutilized significantly for over a century. In order to avoid a similar delay, it is important to persist in the fundamental understanding of these systems and how to process nano-composites with tailored micro-structure for mechanical performance.

**Figure 3:**
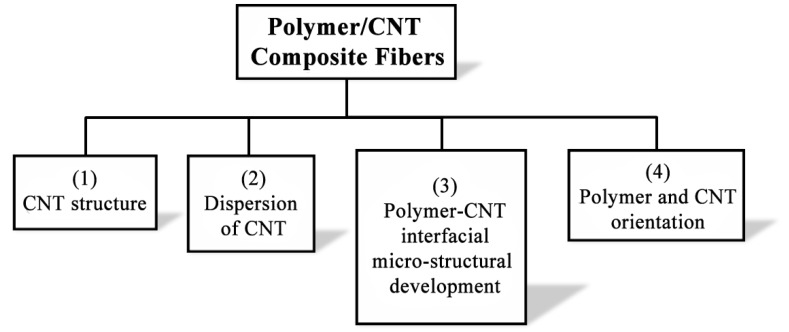
Four major factors affecting the micro-structural development in polymer/CNT composite fiber during processing.

The inherent properties of CNT assume that the structure is well preserved (*i.e.*, large-aspect-ratio and without defects). Going further, the first step toward effective reinforcement of polymers using nano-fillers is to achieve a uniform dispersion of the fillers within the hosting matrix, and this is also related to the as-synthesized nano-carbon structure. Secondly, effective interfacial interaction and stress transfer between CNT and polymer is essential for improved mechanical properties of the fiber composite. Finally, similar to polymer molecules, the excellent intrinsic mechanical properties of CNT can be fully exploited only if an ideal uniaxial orientation is achieved. Therefore, during the fabrication of polymer/CNT fibers, four key areas need to be addressed and understood in order to successfully control the micro-structural development in these composites. These are: (i) CNT pristine structure; (ii) CNT dispersion; (iii) polymer–CNT interfacial interaction; and (iv) orientation of the filler and matrix molecules (Figure 3). This review will highlight some key papers that have focused on these areas as a means to tailor the composite structure and advance the mechanical performance of the polymer nano-composite.

**Figure 4:**
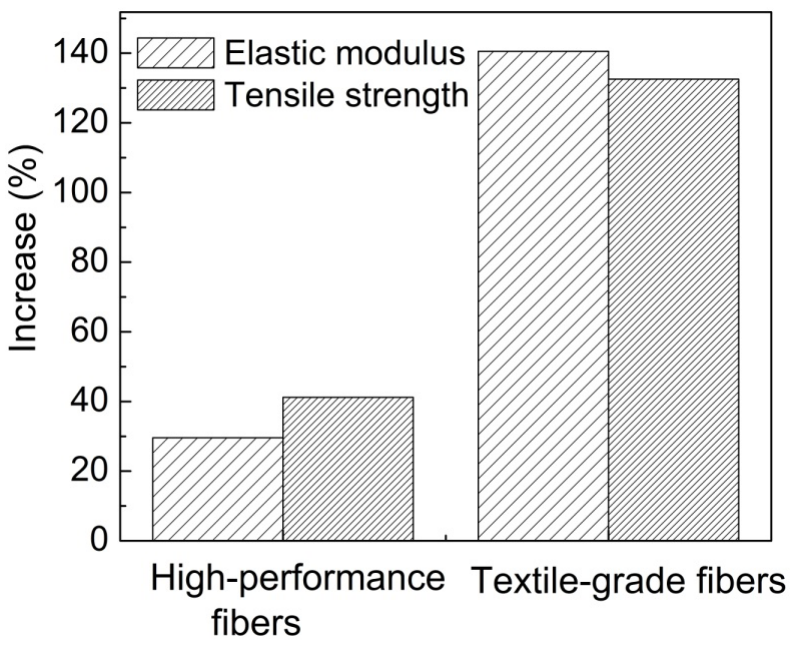
Average percent increase comparison between control fibers (no fillers) and composite fibers for both the Young’s modulus and tensile strength properties. The graph compares the differences in percent increase for the high-performance and textile-grade polymeric fiber materials [35,36,38,39,40,41,42,43,44,45,46,47,112,113,114,115,116].

A further analysis of the published literature also shows an interesting trend, whereby the percent increase in mechanical properties for polymer composite fibers is related to the inherent polymer structure (Figure 4). It is already known that the polymer chain conformation plays a role in the structural development of the fiber, which translates to the composite material, as well. Composite fibers fabricated using aromatic polymer matrices exhibit significantly lower percent increase in mechanical properties as compared to flexible polymer matrices. For rod-like (e.g., aromatic) polymer chains in high-performance fibers, the addition of CNT fillers improves the composite properties mainly due to reinforcement. However, for the more flexible polymer in textile-grade fibers the improvement in mechanical performance is much more pronounced and may be due to factors beyond reinforcement. Such factors include polymer chain orientation, as well as polymer crystallization due to the presence of the CNT in the matrix. This contribution will also outline some of the studies highlighting such phenomena.

### 3.1. CNT Structure and Dispersion

Structural control of CNT graphitic materials is mostly influenced by the synthesis processes, which determines their aspect ratio, morphology and dimensions, crystalline structure, purity, and properties. Presently, a large amount of CNT can be prepared by electric arc discharge [117,118], laser ablation [119] and chemical vapor deposition (CVD) [120] methods. CVD is the most dominant method of large volume CNT production and typically uses fluidized bed reactors that enable uniform gas diffusion and heat transfer to metal catalyst nano-particles [121]. A typical as-synthesized batch of CNT has large tube variations in terms of the type (*i.e.*, metallic *vs.* semiconducting), purity, and structure uniformity (*i.e.*, length, elongated or coiled conformation, tube or bundle diameter, aspect ratio, morphology consistency, and crystallinity). All of these factors influence their electric or thermal conductivity, and mechanical properties [17,18,19,20,21,22]. Contaminants generated during synthesis also influence the final mechanical properties and often require costly thermal annealing and chemical treatment for their removal. These steps can introduce defects in CNT sidewalls and shorten CNT length. Obtaining highly purified and uniform CNT batches is a major challenge that has a significant impact on their use for applications in polymer/CNT fiber composites. The variation in the CNT from batch-to-batch results in significant property variation [122], which ultimately leads to disparities in the properties of the subsequent composite materials [30,31,32].

**Figure 5:**
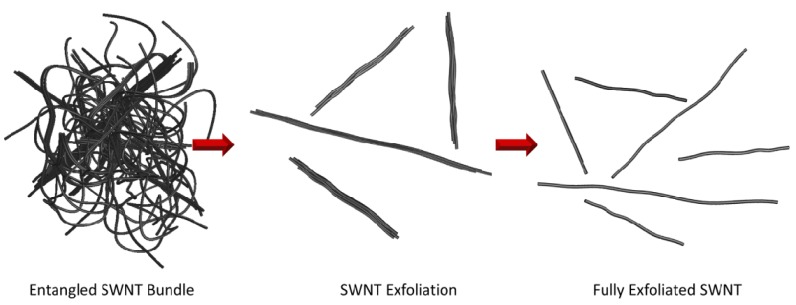
Schematic representing the disentanglement, exfoliation, and length scission processes for SWNT bundles during dispersion.

CNT are characterized by tubes with different wall numbers, for example, SWNT, double-wall carbon nanotubes (DWNT), few-wall carbon nanotubes (FWNT), and MWNT. As-produced and purified nanotubes also possess surface defects and are not geometrically identical (*i.e.*, chirality, diameter, length). For this reason, the actual mechanical strength, as well as other properties, significantly differs from the theoretical predictions. As-synthesized CNT batches are normally randomly oriented and entangled with one another (Figure 5). This is especially the case for SWNT, DWNT, and FWNT. Due to the high specific area, >10^3^ m^2^/g for these nanotubes [123] small attractive forces (*i.e.*, ~0.5 eV) are able to form single CNT contacts that result in bundle formation [124] leading to aggregation. This makes the process of dispersion and separation into individual tubes for uniform distribution into the polymer matrix a major challenge.

Disentanglement and exfoliation processes including centrifugation, homogenization, stirring, and especially ultrasonic dispersion typically result in a sacrifice of the structural integrity and length preservation of the nanotubes (Figure 5). However, both the dispersion in terms of tube bundle size and length are very important to produce polymer composites which fully utilize the CNT fillers. Studies of dispersion effects on exfoliation and length reduction of CNT have been conducted and reported by several research groups [125,126,127,128,129,130]. While the reduction of the CNT length which occurs during dispersion is not always desirable for producing high-performance materials, improving exfoliation is desired and has a significant positive impact on processing [131].

**Figure 6:**
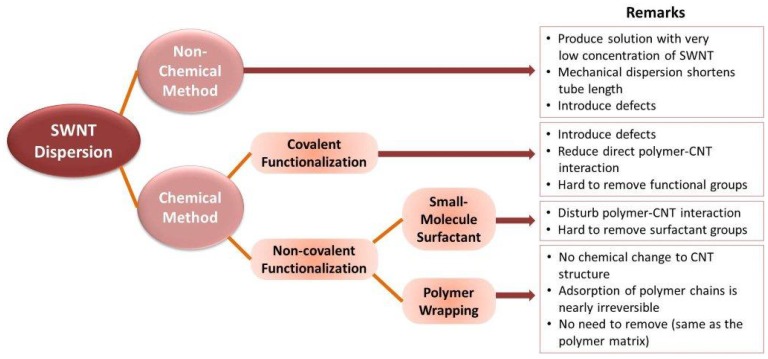
Currently used methods for SWNT dispersion towards fabrication of polymer/CNT nano-composites.

Common dispersing methods that have been utilized are generally the modification on SWNT surfaces by covalent [132,133] or non-covalent treatments [134,135]. Non-covalent dispersants are categorized into small molecules and polymers. Exfoliating SWNT bundles through a polymer-wrapping process has been shown to aid the formation of interphase regions in the composite. A very high degree of exfoliation of SWNT can be obtained with low SWNT concentration (<20 mg/L) in organic amide solvents such as *N*,*N*-dimethylformamide (DMF), *N*-methylpyrrolidone (NMP) and *N*,*N*-dimethylacetamide (DMAc) [136,137,138]. Without chemical treatment, the formation of true solutions can only be obtained at very low concentrations of SWNT (typically below 20 mg/L) [136]. In this case, a minimal enthalpy of mixing close to zero is achieved, indicating athermal solubility [136,139]. Various dispersing methods for SWNT materials are outlined in Figure 6.

**Figure 7:**
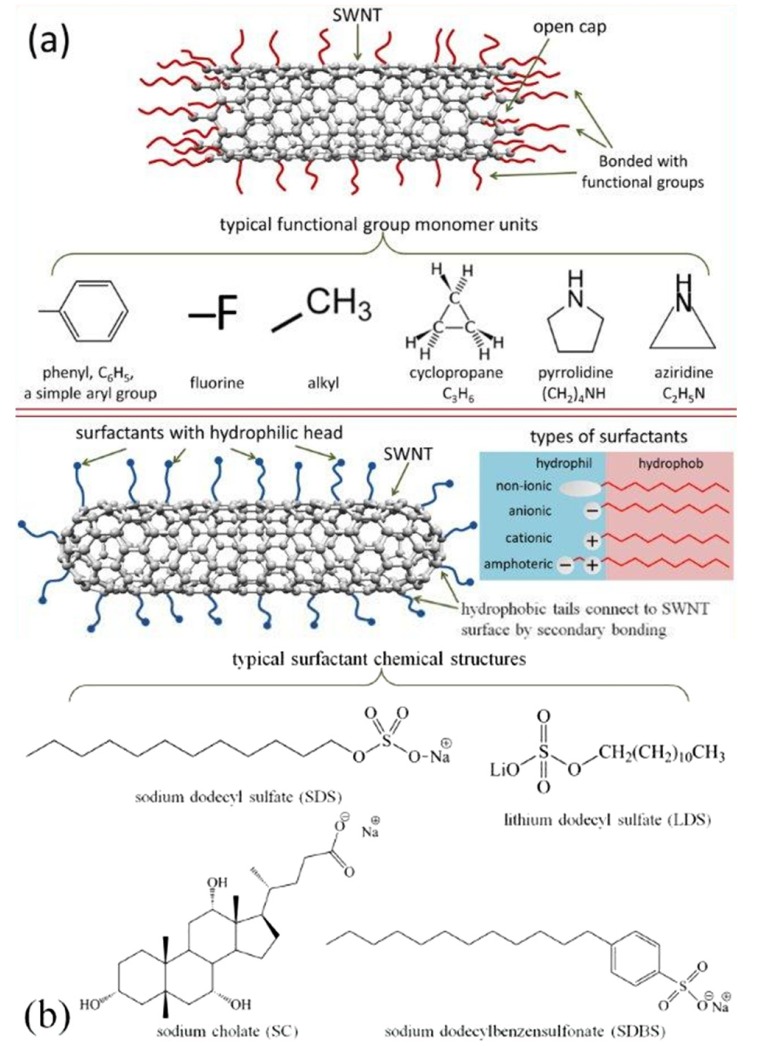
(**a**) Schematic representation of SWNT functionalization by covalent bonding; (**b**) Schematic of SWNT surface modification by small-molecule surfactants.

Covalent functionalization of SWNT has been used to significantly improve the nanotube solubility and chemical compatibility for reinforcing various composite materials. In this process, strong acids or other strong oxidizing agents are used to treat SWNT to create open sites (*i.e.*, break bonds in the graphitic structure) and subsequently attach various functional groups to the open-end and/or defect sites [132] (Figure 7a). Typical methods for covalent functionalization on sidewall include fluorination, ozonolysis, organic functionalization, osmylation, and azomethineylides [132]. Some example additions are aryl (diazonium [140]), fluorine (fluorination [141]), alkyl (radical chemistry [142], Billups reaction [143]), cyclopropane (Bingel reaction [144], dichlorocarbene [145]), pyrrolidine (Prato reaction [146]), and aziridine (nitrene [147]) (Figure 7a). Covalent functionalization has been shown to greatly improve CNT dispersion in polymer matrix [148,149,150,151], and play a critical role in both thermal and electrical properties of CNT/polymer composites [152,153,154]. Chemical functionalization increases the inter-tube contacts (*i.e.*, useful for building up a conductive network) and provides more possibilities to bond the nanotubes to a matrix due to reactive chemical groups. On the other hand, covalent surface treatments can destroy tube structure, resulting in shortening of nanotubes [155,156], creation of defects in the graphitic structure of CNT walls [133,156,157], and in some cases, unzipping of the tube structure. Consequently, chemical functionalization will decrease the mechanical properties of CNT [158]. Non-covalent dispersing methods have also been developed to exfoliate SWNT bundles into individual tubes in different solvents using various anionic, cationic, nonionic surfactants [134,159] (Figure 7b), or polymers [135,160]. The adherence of chemical moieties or polymer molecular wrapping on SWNT surface occurs due to the non-covalent supramolecular interactions, including hydrophobic–hydrophobic interactions, van der Waals forces, π–π interactions, hydrogen bond linkage, and electrostatic attraction [161]. These non-covalent interactions eliminate the chemical modification of the graphitic structure (thus preserving mechanical, electrical, and optical characteristics of the nanotube), and enable the CNT to have improved interactions/solubility in more solvents. 

Dispersion and structure preservation of the nanotube are important to the overall mechanical performance of the composite. As mentioned, in addition to detangling and exfoliation, nanotube length is also sacrificed during dispersion. In the CNT composite, the CNT length is generally on the order of 500 nm to 1 μm. Below a critical length, the CNT cannot transfer its stiffness or strength properties to the polymer matrix, and this results in premature failure of the composite. For this reason, it is recognized that preserving CNT length and structural perfection during composite preparation is also desirable. In an ideal binary bulk composite, the same length registry for the matrix and filler materials is assumed, and this results in an overestimation of the mechanical contributions. To demonstrate the importance of the length contribution of the CNT an example utilizing a composite materials mechanics viewpoint is discussed. An approximate estimation of the CNT’s strengthening mechanism as a function of aspect ratio is determined by using a modified rule-of-mixture (ROM) approach [14,162,163]. Equations (1) to (3) are derived from the ROM approach [14,162,163] and used for this analysis.

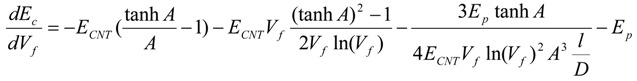
(1)

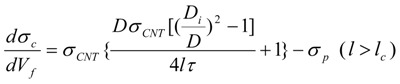
(2)

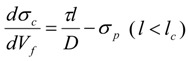
(3)
where A and *l_c_* is a factor given by Equations (4) and (5):

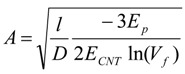
(4)

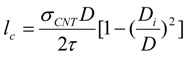
(5)

**Figure 8:**
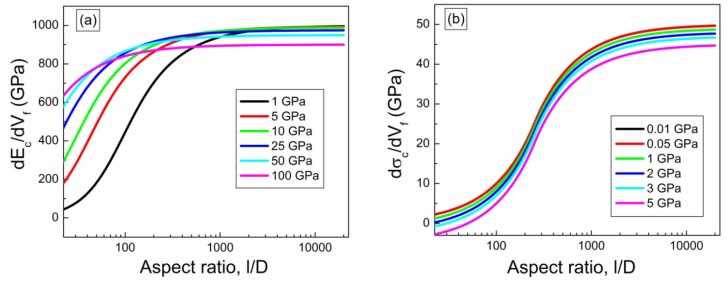
(**a**) Modulus/loading ratio; (**b**) strength/loading ratio as a function of SWNT aspect ratio for composite fibers with full alignment. *E_c_* and *σ_c_* are the composite modulus and tensile strength, *l* and *D* are the length and diameter of the CNT, *V_f_* is the volume of CNT in the composite fibers.

For Equations from (1) to (5) the parameters are defined as follows. *E_c_*, *E_CNT_*, and *E_p_* are the modulus of composite, CNT and polymer matrix, respectively. *l*, *D_i_*, and *D* are the length, interior and exterior diameters of CNT, respectively. *τ* is the shear strength at interphase between polymer and CNT, *V_f_* is the CNT filler loading in the composites, and *l_c_* is the critical length above which strength of CNT can be transferred efficiently to the polymer matrix. The SWNT modulus, strength, and interfacial shear strength are taken to be 1 TPa, 50 GPa, and 100 MPa (*i.e.*, based on computational predictions) [20,164,165], respectively. To demonstrate the importance of the length contribution in the composite, Figure 8 is plotted by using polymer matrix modulus values ranging from 1 to 100 GPa, and strength values ranging from 0.01 to 5 GPa. These values correspond to the typical properties reported for polymers used in CNT composite processing [35,36,38,39,40,41,42,43,44,45,46,47,112,113,114,115,116]. The modulus and strength increase with respect to aspect ratio is shown in Figure 8. It can be seen that both stiffness and strength of the fibers scale with aspect ratio. A similar trend has also been reported for composite films [26].

It is clear that the dispersion of the CNT in terms of exfoliation, distribution, and length preservation are all-important aspects affecting the development of the composite microstructure. Each factor is dependent on the other and finding the right balance remains a challenge. Although several methods for dispersion have been discussed, it is important to recognize that without good polymer nanotube interaction, even well-dispersed CNT may not provide effective reinforcement of the matrix. To improve polymer-CNT interactions, interfacial development is necessary. The following Section 3.2 discusses some of the mechanisms for the development of interfacial structures in the polymer composite fibers.

### 3.2. Interfacial Development in Polymer/CNT Fibers

The interphase can be defined as the three-dimensional boundary between the fiber and the matrix. It has been recognized that control over the interphase (*i.e.*, communication between the matrix and nano-filler) regions will have significant effects on the macroscopic performance of the material [166]. To do this will require deeper fundamental understanding of the nano-composite system in terms of morphology formation during processing. The interfacial interaction occurs through several mechanisms: (i) mechanical coupling, micro-mechanical interlocking and polymer chain-CNT entanglement; (ii) physical interaction, including van der Waals forces, electrostatic forces, or epitaxial crystal formation; and (iii) chemical interactions. As mentioned in the previous section, these chemical interactions include covalent bonding and physical bonding such as surfactant-assisted dispersion of CNT [133], plasma polymerization [167], and polymer wrapping [168,169]. 

Several studies have focused on understanding the strength of the interface for polymer/CNT materials. For PVA/CNT composites, it was found that the shearing resulted in fracture of the matrix before the breakage of the interphase polymer [170]. The shear stress was determined to be around 40 MPa, which is in reasonable agreement with predicted values of ~50 MPa [170]. Other computational works have also been carried out to predict the interfacial shear stress (IFSS). Polymer systems such as polystyrene (PS) [171], epoxy [172], poly(m-phenylenevinylene-*co*-2,5-dioctyloxy-*p*-phenylenevinylene) (PmPV), and poly(phenylacetylene) (PPA) [173] have been calculated using molecular dynamics, where the calculated IFSS was dependent on both the polymer and CNT. In such cases the IFSS values ranged from 18 to 186 MPa.

Apart from the calculations and simulations, direct measurements have also been reported. The techniques and devices for these measurements include scanning electron microscopy (SEM) [20], transmission electron microscopy (TEM) [174], atomic force microscopy (AFM) [164,175], and scanning probe microscopy (SPM) [176]. These reported values range from 0.02 to 500 MPa [39,116,126,165,174,176]. The larger IFSS values are consistent with composites where covalent bonding is present at the interphase (*i.e.*, functionalized CNT). Values of 0.5 GPa estimated by Wagner *et al.* [174], and 0.35 GPa measured by Cooper *et al.* [176] were measured utilizing the AFM and were attributed to covalent bonding between CNT and polymer. To date, the majority of interphase measurements and predictions have focused on either pristine CNT or functionalized CNT embedded in amorphous polymer melts. Less is known about the interfacial mechanical properties of crystalline polymer at the CNT interphase, especially in cases where the polymer is able to form ordered phases along the CNT length.

Several recent papers have highlighted the importance of crystalline interphase formation in these composites [39,42,43,177,178,179]. It has been observed that CNT can nucleate and template the growth of ordered polymer crystals in several polymer systems including PE [180,181,182,183,184,185], nylon 6,6 [182], PVA [186], PAN [187], poly(butylene terephthalate) (PBT) [188,189,190], isotactic polypropylene (iPP) [191], poly(l-lactide) (PLLA) [191], poly(e-caprolactone) (PCL) [192], and polyethylene-*b*-poly(ethylene oxide) (PE-*b*-PEO) block copolymer [193]. One of the dominant reinforcement mechanisms in polymer/CNT composites has been suggested to be the presence of ordered polymer interfacial coating structure near CNT [194]. This ordered structure is able to form due to the ability of CNT to interact specifically with the polymer matrix. Ordered or crystalline polymer structure in polymer nano-composites is mechanically stronger than amorphous structure due to the presence of fewer defects or less disordered regions. Therefore, it is important to study CNT-induced polymer crystallization to control these mechanisms during the formation of the interphase in the polymer/CNT composites.

On a molecular level, a decreased interpenetration/entanglement of chains near a solid interface cause chain configuration, the configuration–change energies, and repeat unit-surface interaction energies to change [195]. In addition, changes in reaction kinetics and interfacial mobility (*i.e.*, due to crosslink density) can also affect the system [195]. Glass transition, polymer diffusion, nanotube diffusion, crystalline structure, crystallization kinetics, and properties can also be altered [195]. This phenomenon is not seen with other commonly used micro-scale fillers [195]. Additional work has shown that the interphase polymer morphology is completely different from the bulk polymer in the composite, and this translates to high modulus and tensile strength values (*i.e.*, modulus between 5 and 400 GPa and strength >1 GPa). Examination of these interphase regions by microscopy shows that they display crystalline perfection [42,43,44,178].

**Figure 9:**
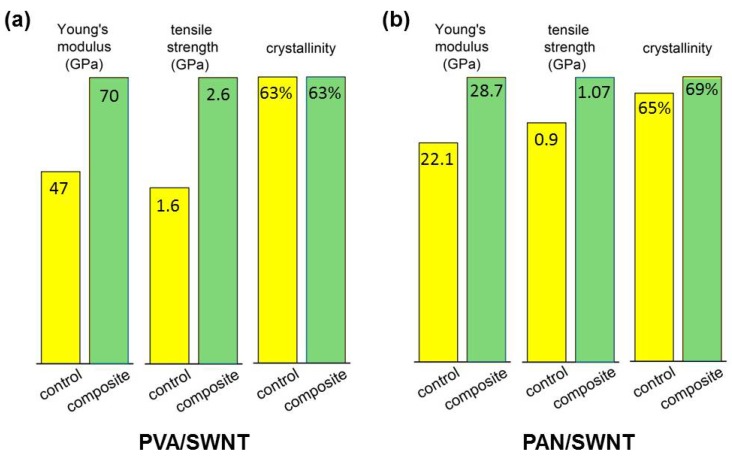
Comparison of mechanical property improvement for (**a**) poly(vinyl alcohol) PVA/CNT and (**b**) polyacrylonitrile (PAN)/CNT fibers, which display SWNT-templated polymer extended-chain crystallization and orientation [42,178].

As previously mentioned, several works have also shown the ability of the nanotube to nucleate polymer crystal growth at the interphase [182,196], as well as template crystal growth and orientation in polymers [42,43,177,178,181,185,197]. This templating effect of CNT in polymer composites has been proven to have an effective contribution toward the stress transfer mechanism of load between the polymer matrix and filler [42,179] (Figure 9). In such cases where templated interphase structure was found to be present at the interphase, the mechanical properties for the composite were significantly increased. It is also interesting that the overall crystallinity value for the composite as compared to the control fibers is relatively the same. This implies that while a portion of the matrix polymer forms a highly ordered interphase structure the bulk-matrix remains semi-crystalline and relatively disordered. It is also worth mentioning that the increase in mechanical properties does not follow rule-of-mixture predictions. This is due to the contribution from the interphase polymer, which is often unaccounted for. Several recent works have attempted to include this contribution for better understanding of the composite micro-structural contribution to the bulk properties [40,61]. It is also important to note that in some CNT-polymer systems where CNT templating is found, the crystallinity is typically much higher in the composite *versus* the control system. In such cases, the effect of templating alone is difficult to assess. Here, the focus is on two systems, which display similar crystallinity in order to understand the role of the template-oriented polymer interphase contribution.

It has also been recognized that in cases where the interphase regions are not template or oriented (*i.e.*, showing chain disorder), the mechanical enhancement is not that significant [198]. Interfacial stress transfer is a critical component/parameter controlling the performance of the composite. Complete stress/load transfer from the polymer to the nano-filler is achievable if there is strong adhesion. Based on these high-resolution transmission electron microscopy (HR-TEM) studies, better chain packing was also shown to exist at the interphase [42,43,181].

**Figure 10:**
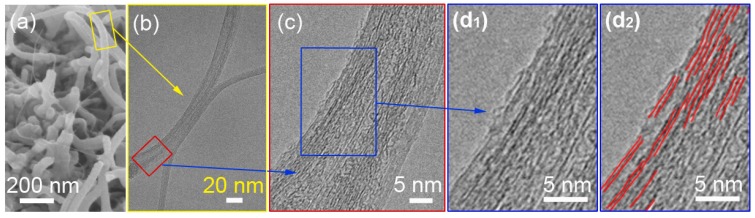
(**a**) Scanning electron micrograph (SEM) of PAN tubular coating on SWNT. High-resolution transmission electron micrograph (HR-TEM) of tubular coated PAN/SWNT samples; (**b**) at the onset of electron beam exposure; (**c** and **d_1_**) show an area of the PAN/SWNT sample where the PAN lattice of ~0.52 nm is observed; and (**d_2_**) a schematic highlighting the PAN lattice observations in (**d_1_**) [187].

Recently shear crystallization studies in hybrid polymer/SWNT dispersion were used to induce oriented polymer crystallization in the presence of the SWNT. These studies were specifically focused on developing a procedure for producing ordered interphase structure on the CNT. Figure 10 shows a HR-TEM image for a PAN-SWNT interphase, where the polymer extended-chain morphology has been templated by the nanotube [187]. These fundamental crystallization studies provide good insight toward the morphological capabilities of the polymer influenced by this mechanism. In terms of processing polymer/CNT composite materials, these crystallization processes may even be incorporated into fabrication procedures.

The fundamental understanding of the interfacial relationship between the polymer and CNT is still being developed. However, it is recognized that this micro-structural development is necessary to improve stress-transfer mechanisms in the composite. Polymer crystalline interphase structures have very interesting behavior in terms of composite failure mechanisms [40,199] and properties [42,43,44,45]. What is lacking is the fundamental understanding for consistently producing such interphase structures during processing of the composite. Most of the studies highlighted have recognized the existence of this micro-structural development only after the composite is being characterized. It is important that such interfacial structures are pursued during composite preparation/fabrication, and this requires that material design approaches be incorporated into such steps. To do this will require better understanding for the development of the polymer interface, which is dependent on many processing factors. However, understanding this rather complex problem may have tremendous implications toward producing polymer/CNT composite fibers that can finally begin to consistently approach the long heralded predicted values.

### 3.3. Orientation and Alignment Effects

The importance of inducing extended-chain polymer crystal growth and orientation in these nano-composite materials is understood when looking at the theoretical modulus calculations for various flexible polymer systems (*i.e.*, the modulus calculations are based on the polymer being in the extended-chain conformation) [200,201]. Thermoplastic polymers like PE, cellulose, poly(tetrafluoroethylene) (PTFE), and PVA all possess Young’s modulus ranging from 100 to 250 GPa along the axial direction [202]. Similarly, the predicted tensile strength of a perfect or fully extended polymer fiber is potentially on the order of ~30 GPa [203]. To date, the majority of thermoplastic polymeric materials possess low crystallinity ~30% to 40% and poor orientation leading to low mechanical properties (*i.e.*, stiffness and strength <10 GPa and ~0.5 GPa, respectively). As outlined, the introduction of CNT as a templating material for extended-chain crystallization and orientation has provided a new route to tailor polymer morphology in a fiber [178,197]. Several studies have shown the ability of these nano-carbon fillers to modify and influence the morphology of the polymer in the composite [42,178,204]. These nucleation, crystallization, and orientation effects are especially observed in composites with low nano-carbon loading (<1 wt %), and have a significant impact on the overall structure and properties of the composite material [42,43].

**Figure 11:**
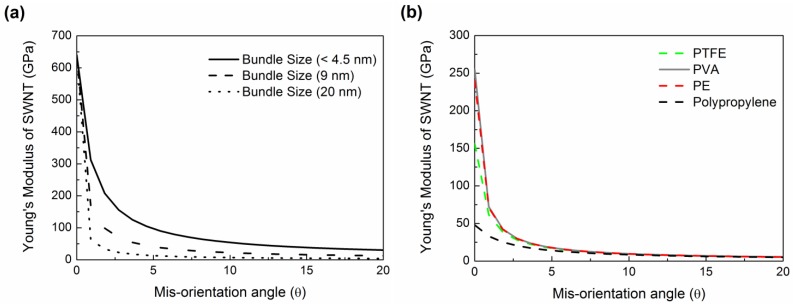
Graph showing the effect of mis-orientation on the effective Young’s modulus for both (**a**) SWNT fillers and (**b**) various linear polymers.

Alignment of CNT or CNT ropes is another important factor in determining the mechanical properties of composites containing them. According to the continuum mechanics calculations, the moduli of both SWNT filler and polymer chains along the axial direction drop abruptly for only slight mis-orientation with respect to the fiber axis (Figure 11). For SWNTmaterials, this effect is less pronounced as the SWNT bundle diameter decreases [205] (Figure 11). The effect of orientation on modulus properties for anisotropic composites can be taken into account using Equations (6) and (7) [205].

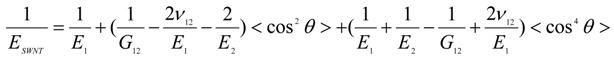
(6)

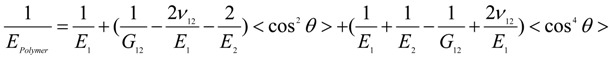
(7)
where, the values of 〈cos^2^ *θ*〉 and 〈cos^4^ *θ*〉 are given by Equations (8) and (9) [206]:

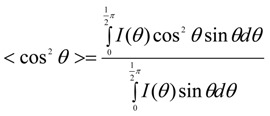
(8)

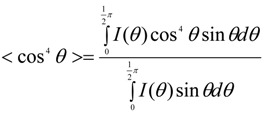
(9)

The parameters used for the orientation analysis (Figure 11) are provided in Table 3. What is immediately obvious is that in the polymer/CNT composite fiber, the full alignment of the polymer chain and the CNT is paramount. This is not an easy task. To date, only a handful of polymer-based high-performance fibers exists (*i.e.*, Kevlar^®^, Spectra^®^, Zylon^®^), and this is due to the high chain alignment in the micro-structure either afforded by the inherent polymer conformational structure (*i.e.*, rod-like molecules—Kevlar^®^ and Zylon^®^) or special processing of low concentration polymer solutions to reduce chain entanglement (*i.e.*, gel-spinning of polyethylene—Spectra^®^). However, in more recent work, the similarities between polymers and CNT, CNT templating effects, CNT liquid crystalline nature, and the ability of nano-carbons materials to lubricate polymers during alignment have been recognized. These factors all have significant implications toward greatly improving polymer chain alignment during processing of the composite.

**Table 3:** List of parameters used for Equations (6) and (7) for orientation analyses corresponding to the Young’s modulus contribution along the axial direction [202,207,208].

Parameters	E_1_ (GPa)	E_2_ (GPa)	ν	G_12_ (GPa)
SWNT				
20 nm bundle	1000	15	0.17	0.7
9 nm bundle	1000	15	0.17	2.3
<4.5 nm bundle	1 000	15	0.17	6
Polymers				
Poly(vinyl alcohol) (PVA)	255	9	0.338	1
Polyethylene (PE)	240	4.3	0.46	1
Poly(tetra fluoroethylene) (PTFE)	156	5	0.46	1
Polypropylene (PP)	42	2.9	0.45	1

By comparing the structure, properties, phase behavior, rheology, processing, and applications between SWNT and rigid-rod polymers, SWNT are considered as polymeric materials [209,210]. As mentioned, the similarity between CNT (especially SWNT) and polymers will allow the polymer chains to interact with SWNT more readily and nucleate on SWNT surfaces due to epitaxy. For this reason, SWNT are potentially able to align the chains parallel to the axis direction and template polymer crystallization with extended-chain conformation. For polymeric materials extensional force (usually conducted through shear flows in melt or solution) is required for inducing the extended-chain crystallization and the subsequent growing of the bundle-like fibrils or shish-kebab structures [211,212,213]. This shearing mechanism is also needed to grow fibrillar (extended-chain) crystals in polymer/CNT hybrid systems [42,43,178,181]. The processing of extended-chain polymer crystals in CNT systems is difficult and not as typical as the observation of folded-chain crystal structures in these composites. However, a few previous works have shown that SWNT can induce nucleation of extended-chain crystallization and template the alignment of polymer chains in PE [181], PBT [214], poly(ethylene terephthalate) (PET) [177], PAN [43,44,45], and PVA [42,178] systems. The presence of CNT is considered to largely contribute to the polymer nucleus size in the hybrid system, which suppresses the energy barrier for fibrillar crystallization by providing sufficient heterogeneous nucleation sites due to epitaxial interaction [85].

Under quiescent conditions, the final crystalline structure and morphology are determined by the filler characteristics (*i.e.*, concentration, composition, filler size, and shape) and by the interaction between the filler and the polymer matrix. In the presence of the shear flow, the influencing effects extend to shear rate, shear duration, and the interaction between shear and fillers [213]. In a polymer/nano-particles hybrid system, the introduction of nano-fillers and polymers into shear flow has been shown to create a synergistic effect for promoting crystallization, due to the changes in the local stress levels and orientation of chains surrounding the nano-particles upon the application of shear [213,215,216]. For this reason, the rod-like CNT can greatly induce anisotropic nucleation sites at the interphase and promote the subsequent crystal growth in the flow direction.

Under appropriate shear flow at a specific crystallization temperature, PE and PAN have been shown to crystallize into extended-chain shish directly on SWNT [181,187] surface, followed by nucleation of folded-chain lamellae. Based on the small-angle X-ray scattering (SAXS) analysis for the pure PBT system and PBT/SWNT composites, it was shown the very low SWNT loading (0.2 wt %) can largely template the morphology of crystallization during flow, providing a method to obtain a highly desirable fiber-like morphology [214]. Patil *et al.* have concluded that within the sheared PE/CNT nano-composite system, the presence of CNT significantly promote the polymer chain orientation, the length increase, and the stability of the hybrid shish-kebab structures, due to CNT templating chain alignment as compared to the sheared pure PE system [217,218,219]. Wide-angle X-ray diffraction (WAXD) studies on drawn PET/SWNT composite showed that oriented crystallization of PET was induced by aligned SWNT in a randomized PET melt [177]. This orientation of the PET survived even after re-melting [177]. No orientation was observed in the re-melting process in the neat PET system, indicating the templating role of SWNT upon shear for polymer crystallization [177]. These studies demonstrate the synergistic effects of the presence of SWNT and shear flow on promoting polymer extended-chain crystallization at the interphase in the nano-composites.

**Figure 12:**
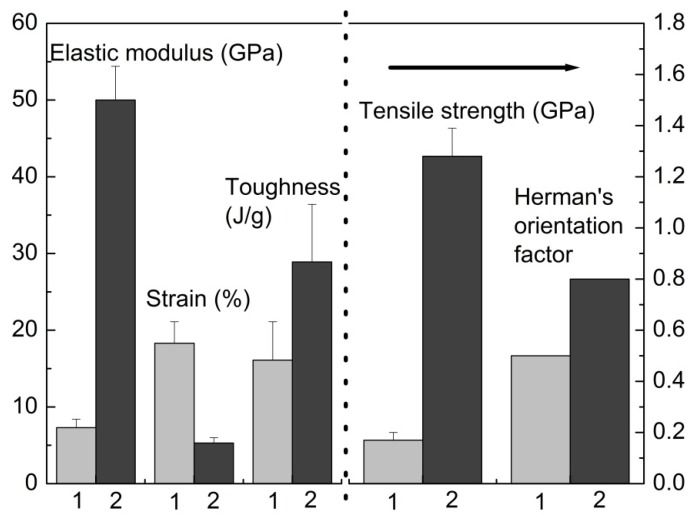
Bar chart comparing modulus, strain, toughness and tensile strength (1 is the control fiber and 2 is the composite fiber) for drawn control and composite PVA/SWNT fibers [61].

In addition to templating, the use of rigid nano-carbons in polymer matrices may also enable increased polymer chain alignment during processing [61]. Improvement in chain alignment has been reported where an orientation factor (*f*) increase from 0.5 to 0.8 was found. This subsequently led to a drastic increase in the mechanical performance of the composite as compared to the control fiber (Figure 12). This work demonstrates the ability to use unique nano-fillers to act as a lubricant during drawing to facilitate polymer chain extension and orientation.

Several studies have shown that the polymer chains form preferential alignment in the presence of CNT, and this is not the case in their absence [61,177,178,181,214]. What is needed at this point is the understanding of how to take advantage of such a phenomenon during processing of the composite. The unique similarities between the CNT and polymer [210] may afford opportunities to develop new special processing techniques that can take advantage of such parallels to produce high-performance polymer/CNT fibers with well-controlled micro-structures.

## 4. Prospects and Challenges for Processing Polymer/CNT Composites with Controlled Structural Development

This contribution has outlined studies that recognize that tailoring interfacial properties in materials has a direct influence on the overall performance of the system. This is especially true for composite materials, which are composed of two or more dissimilar materials. Without good interaction between the components of the system, the contribution from each is diminished. To date, the introduction of nano-materials and their use in composite systems have shown that these filler materials can have tremendous impact on the matrix components even without any optimization. However, the majority of these improvements have so far been incremental. Taking full advantage of the CNT material requires more design as it pertains to the interaction between the filler and the matrix, dispersion processes, and alignment of this hybrid system during fiber spinning. For this reason, future-processing approaches of polymer/CNT materials should incorporate some modeling/computational aspects in order to predict what kind of effects these parameters may actually have on the polymer and nano-filler. This task is a challenge in that to understand such a procedure, a truly multi-scale approach is necessary to envision all steps from the atomic/nano-scale (*i.e.*, polymer chain and CNT), to the macro-sale spinning procedure. In addition, the complexity of modeling polymer solutions and melts in the presence of these nano-carbons as they form solid fibers renders it a difficult task.

Experimentally, as highlighted in this contribution for many polymer/carbon nanotube composites, it has been demonstrated that the polymer is able to have some direct interaction with the nanotubes. For specific systems, the polymer has a high affinity for wetting, wrapping, or even crystallizing in and around the nanotubes. What is attractive about polymer crystallization in the presence of the CNT is the ability to create ordered polymer structures in the vicinity of CNT, with implications for a wide-range of applications. Thus far, these phenomena have mainly been observed. Therefore, fundamental studies are necessary to truly understand the inherent ability of CNT to nucleate and template polymer crystallization, and its effects on the ordered conformation characteristics of polymers. Such processes are influenced by the polymer molecular architecture, chemical make-up, conformational capabilities, as well as nanotube diameter, type (*i.e.*, MWNT, SWNT, FWNT), graphitic perfection, and chirality. The determination and control of these parameters are required to induce the crystallization process, whether processing fibers from the melt or solution.

It is clear that both the experimental and modeling challenges are very important for design processing fabrication approaches that can truly make the most of the polymer/CNT hybrid system in terms of structural development and ultimate properties. Going forward in this field will require such approaches to achieve the dream that began almost two decades ago.

## 5. Conclusions

This review summarizes studies on the various parameters that affect the strengthening mechanisms in polymer/CNT fiber composite systems as a function of processing. CNT containing polymeric fibers have exhibited improved mechanical and physical properties such as tensile strength, Young’s modulus, strain-to-failure, toughness, and resistance to molecule changes from both solvent and heat treatments. Experimental factors influencing composite processing include CNT structure, dispersion, interfacial interaction, and alignment/orientation of polymer chains and CNT. The combination of these factors needs to be well controlled in order to optimize the resultant mechanical properties of the bulk composite fiber. An understanding of these factors is complex and a great challenge in the field of nano-composite processing. However, increasing fundamental experimental insight coupled with computational and “materials by design” approaches will lead to more efficient use of CNT in composites and better optimization of fabrication procedures.
